# Recent Advances in Fluorescent Nanoparticles for Stimulated Emission Depletion Imaging

**DOI:** 10.3390/bios14070314

**Published:** 2024-06-21

**Authors:** Liqing Qi, Songlin Liu, Jiantao Ping, Xingxing Yao, Long Chen, Dawei Yang, Yijun Liu, Chenjing Wang, Yating Xiao, Lubin Qi, Yifei Jiang, Xiaohong Fang

**Affiliations:** 1 Academy of Medical Engineering and Translational Medicine, Tianjin University, Tianjin 300072, China; 1020235012@tju.edu.cn; 2 The Key Laboratory of Zhejiang Province for Aptamers and Theranostics, Zhejiang Cancer Hospital, Hangzhou Institute of Medicine (HIM), Chinese Academy of Sciences Hangzhou, Hangzhou 310022, China; liusl21@mail.ustc.edu.cn (S.L.); pingjt@qlu.edu.cn (J.P.); 221122250264@zjut.edu.cn (X.Y.); 201806021103@zjut.edu.cn (L.C.); yangdawei@him.cas.cn (D.Y.); liuyj595mt@163.com (Y.L.); wangchenjing@him.cas.cn (C.W.);; 3 School of Chemistry and Materials, University of Science and Technology of China, Hefei 230026, China; 4 College of Materials Science and Engineering, Zhejiang University of Technology, Hangzhou 310014, China; 5 School of Molecular Medicine, Hangzhou Institute for Advanced Study, Hangzhou 310024, China; 6 Institute of Chemistry, Chinese Academy of Sciences, University of Chinese Academy of Sciences, Beijing 100190, China

**Keywords:** super-resolution imaging, STED, fluorescence probes, fluorescence nanoparticle, luminescent materials

## Abstract

Stimulated emission depletion (STED) microscopy, as a popular super-resolution imaging technique, has been widely used in bio-structure analysis and resolving the dynamics of biological processes beyond the diffraction limit. The performance of STED critically depends on the optical properties of the fluorescent probes. Ideally, the probe should process high brightness and good photostability, and exhibit a sensitive response to the depletion beam. Organic dyes and fluorescent proteins, as the most widely used STED probes, suffer from low brightness and exhibit rapid photobleaching under a high excitation power. Recently, luminescent nanoparticles (NPs) have emerged as promising fluorescent probes in biological imaging due to their high brightness and good photostability. STED imaging using various kinds of NPs, including quantum dots, polymer dots, carbon dots, aggregation-induced emission dots, etc., has been demonstrated. This review will comprehensively review recent advances in fluorescent NP-based STED probes, discuss their advantages and pitfalls, and outline the directions for future development.

## 1. Introduction

Exploring biological systems at the nanoscale yields profound insights into the composition and functionalities of different bio-structures, shedding light on the intricate workings of various physiological and pathological processes [1,2]. Optical fluorescence microscopy, in particular, has emerged as a pivotal tool in this endeavor due to its ability to visualize samples in their native state with minimal invasiveness compared to electron microscopy techniques like scanning electron micrograph [3,4,5] and transmission electron microscope (TEM) [6,7,8,9]. However, the conventional fluorescence microscopies, including confocal laser scanning microscopy (CLSM) [10,11,12] and total internal reflection microscopy [13,14,15], are constrained by Abbe’s diffraction limit, which restricts their ability to resolve objects closer than approximately 200 nm. To overcome this fundamental limitation, super-resolution microscopy techniques have been developed. These advanced methodologies, including Stimulated Emission Depletion (STED) microscopy [16,17,18,19], Structured Illumination Microscopy [20,21,22,23,24], Stochastic Optical Reconstruction Microscopy [25,26,27,28], Photoactivated Localization Microscopy [29,30,31,32], Super-Resolution Optical Fluctuation Imaging [33,34,35,36], Point Accumulation for Imaging in Nanoscale Topography [37,38,39,40], and others [41,42,43,44], push the boundaries of optical imaging beyond the diffraction limit. Among these techniques, STED microscopy stands out as it combines high spatial resolution with a decent imaging speed. STED microscopy enhances resolution solely through optical means, which allows for the rapid acquisition of thousands of images without extensive data analysis, making it an invaluable tool to analyze biostructures and dynamics at the nanoscale.

The core of STED microscopy lies in the manipulation of the point spread function (PSF) in laser scanning microscopy. This manipulation is achieved through the superposition of a high-intensity donut-shaped depletion beam, referred to as the STED light, with an excitation laser. Strategically positioned in the lower-intensity region at the long wavelength of the emission spectrum, the STED light ensures minimal self-excitation. It selectively quenches fluorescence by reverting excited dye molecules to their ground state, thereby enabling the detection of fluorescence solely from the centrally excited dye molecules within the focus [16]. The STED light is achieved via optical vortices with helical phase distributions, which trigger stimulated emission in fluorophores [45]. STED microscopy attains sub-diffraction-limit spatial resolution by selectively capturing fluorescent signals from the donut’s center. The achieved resolution is mathematically expressed as $D=\frac{\lambda }{2NA\sqrt{1+I_{sTED}/I_{sat}}}$, where λ denotes wavelength, *NA* is the numerical aperture, *I_sted_* represents STED laser power, and *I_sat_* signifies the saturation of the stimulated emission, governed by excitation parameters and fluorophore characteristics [45]. Notably, the achieved resolution is directly correlated with the intensity of the depletion beam. Consequently, ideal STED imaging probes must exhibit a distinct set of optical properties, encompassing high brightness, good photostability, and a keen sensitivity to the depletion beam. Additionally, probes must possess a diminutive size, excellent biocompatibility, high labeling density, and homogeneity to ensure their optimal performance in STED microscopy [46,47,48].

**Figure 1:**
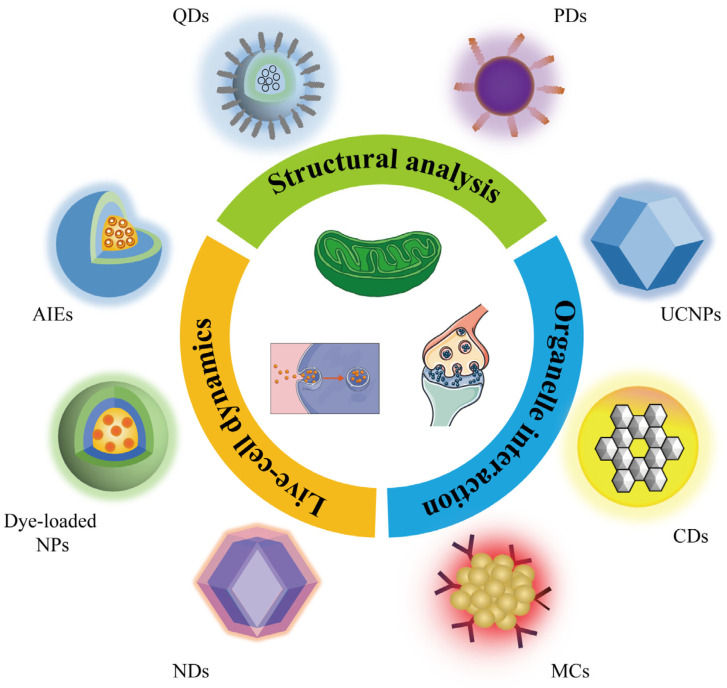
Schematic illustration of the various FNPs and biomedical applications of STED microscopy.

Traditionally, organic dyes and fluorescent proteins have been employed as STED probes; however, they often suffer from limitations such as low brightness and rapid photobleaching under high excitation powers. Recently, there has been a surge of interest in luminescent nanoparticles (FNPs) as alternative fluorescent probes due to their superior brightness and photostability [49,50,51]. Various types of FNPs, including quantum dots (QDs), polymer dots (PDs), carbon dots (CDs), upconversion nanoparticles (UCNPs), aggregation-induced emission dots (AIEs), metal clusters (MCs), nanodiamonds (NDs), and dye-loaded nanoparticles (NPs), have been explored for their potential as STED imaging agents (Figure 1). These FNPs exhibit exceptional optical properties, which allow for a better spatial resolution, longer imaging duration, and lower photo-toxicity to be achieved in STED imaging. For example, a single nitrogen-vacancy (NV) center in an individual ND achieved a remarkable resolution of 9.5 nm [52]. QDs750 demonstrates remarkable photostability, capable of generating over 1000 frames of STED images [53]. Furthermore, UCNPs significantly reduce the laser intensity requirements for optical depletion to below 10 mW cm^−2^, resulting in a significantly lower photo-toxicity [54]. A variety of biological systems, including the cytoskeleton, lysosome, nucleus, nucleolus, endosome, endoplasmic reticulum, chromosomes, RNA network in single cell nucleus, and many proteins, have been labeled with FNPs and studied with STED imaging, providing a high level of detail about their structures and dynamics [46,47,48,49,50,51]. In this review, we comprehensively summarize the recent advancements in the field of FNP-based STED probes. We discuss their advantages and limitations (Table 1), focusing on their inherent characteristics and how they influence imaging performance. Finally, we outline potential future directions for the development of FNPs in biological imaging applications, emphasizing their potential to further revolutionize our understanding of life sciences at the nanoscale.

## 2. Recent Advance in FNPs for STED Imaging

### 2.1. QDs

**Figure 2:**
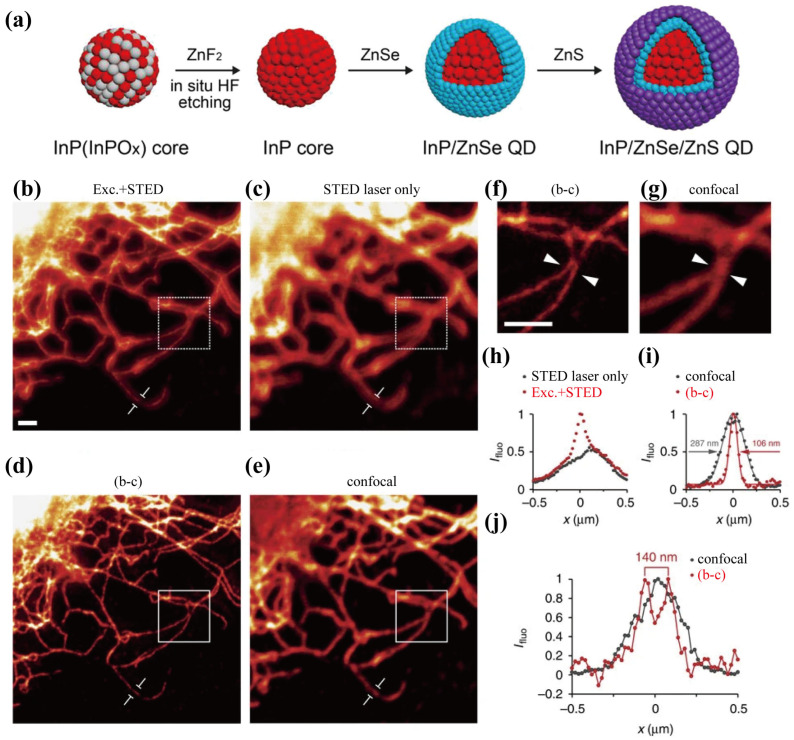
(**a**) Schematic illustration for representative synthetic route of QDs. Adapted with permission from [55]. (**b**) Representative STED microscopy image of Qdot705-labelled Vimentin fibers in REF cells obtained with excitation plus the STED laser beam. Scale bar is 1 μm. (**c**) Image of QDs acquired with the STED laser beam only. (**d**) Processed image obtained by subtraction of the STED laser beam-only image (**c**) from the excitation plus the STED laser beam image (**b**) (3 × 3 median filter). (**e**) The image of same region captured by confocal microscope. (**f**,**g**) Images of the region of interest indicated by white squares in (**d**,**e**), respectively. Scale bar: 1 μm. (**h**) Intensity profile for a single vimentin fiber along the arrow shown in (**b**,**c**). (**i**) Intensity profiles for the subtracted and confocal images indicated in (**d**,**e**), respectively. (**j**) Line profile of the interest position denoted by arrow heads in (**f**,**g**). The single blurred object observed by the confocal microscope was resolved into two fibers with a distance of 140 nm in the super-resolved image. Adapted with permission from [53].

QDs are nano-crystalline semiconductors (Figure 2a), which effectively confine both electrons and holes within their three-dimensional spatial boundaries, thereby exhibiting pronounced quantum-confinement effects [55,56]. These nanoscale materials have garnered considerable attention in recent decades owing to their distinctive photo-physical properties, which diverge significantly from those of bulk semiconducting materials. In biomedical research, QDs have emerged as innovative fluorescent probes, offering a range of advantageous optical characteristics when compared to traditional organic dyes or fluorescent proteins. Specifically, QDs are characterized by a broad absorption spectrum, a narrow and tunable emission spectrum that can span from visible to near-infrared (NIR) wavelengths, exceptional brightness, a prolonged fluorescent lifetime, and good resistance to photobleaching. A notable aspect of their emissions is that the emitted color is not solely determined by their chemical composition but is also closely correlated with the size of the NPs. Larger QDs tend to emit light towards the red end of the spectrum, while smaller particles emit in shorter wavelengths. Furthermore, QDs surface modification typically involves the employment of amphiphilic molecular coatings or the incorporation of functional compounds via an exchange strategy, enabling them to perform biological functionalization, facilitating their utilization in a range of biomedical applications [57]. Capitalizing on these benefits, researchers have developed QDs as probes for super-resolution techniques such as STED microscopy, aiming to achieve long-term imaging with enhanced resolution and stability [58,59].

The Hell group first reported using commercially available ZnS-coated CdSe QDs705 and CdTe QDs as molecular probes in STED imaging [53]. Figure 2b–g demonstrates that the QDs705 (~12 nm) exhibited an improved resolution of ~50 nm with a 775 nm doughnut-shaped STED beam compared to the 230 nm resolution achieved by confocal imaging at the single-particle level, representing a significant 4.2-fold enhancement. Next, they employed QDs705 for the immunofluorescence staining of cellular vimentin fibers, subtracting the background signal solely using the STED beam. This approach achieved a full-width half maximum (FWHM) of 106 nm for primary and secondary antibodies labeled vimentin fibers, whereas the confocal mode yielded an FWHM of 287 nm. Additionally, an intriguing discovery of this work was that when QDs are simultaneously excited and depleted, the photo-blinking behavior of QDs is suppressed, which allows for over 1000 consecutive STED images to be recorded, demonstrating the potential of ultra-long-duration STED imaging. The Qu group investigated the use of highly photostable and luminescent perovskite CsPbBr3 NPs for STED microscopy [60]. These CsPbBr3 QDs demonstrated no attenuation of the fluorescence signal after a 200 min exposure to a 39.8 mW STED beam and exhibited a low saturation intensity of 0.4 mW. The lateral resolution at the single-nanoparticle level reached 20.6 nm, representing a tenfold improvement compared to confocal microscopy. In another study, they used CdSe@ZnS QDs for STED microscopy and observed a low saturation intensity of 0.61 mW and achieved a lateral resolution of 21 nm [61].

As discussed above, QDs are one of the most widely used FNPs for STED imaging and have delivered promising performances. Nonetheless, the inherently broad excitation spectrum of QDs makes them prone to re-excitation by the STED beam, ultimately giving rise to subtle yet observable background patterns upon sole excitation by STED light. QDs also pose challenges pertaining to their limited water solubility and intricate surface modification processes. Although a few studies have explored their toxicity in biological systems, a comprehensive understanding of their impact and underlying mechanisms remains elusive. Hence, a careful evaluation of their biocompatibility is required for in vivo STED imaging [62].

### 2.2. PDs

PDs have gained popularity in bioimaging due to their unique optical properties. PDs are formed by the folding of semiconducting polymers, which can range from a few to hundreds of nanometers in size [63]. Due to the conjugated molecular backbones and the densely packed fluorophores at the nanoscale, PDs exhibit high brightness, good photostability, and efficient energy transfer within NPs [64]. Through doping with PDs with small number of energy-accepting fluorophores, the emission spectra of PDs can be changed efficiently, which allows for PDs of various emission colors to be obtained. PDs also exhibit an excellent biocompatibility and facile bioconjugation. Through co-precipitation with amphiphilic polymers, different functional groups can be introduced to the PDs’ surface, which allows for conjugation with targeting molecules and the labelling of biological structures [65].

**Figure 3:**
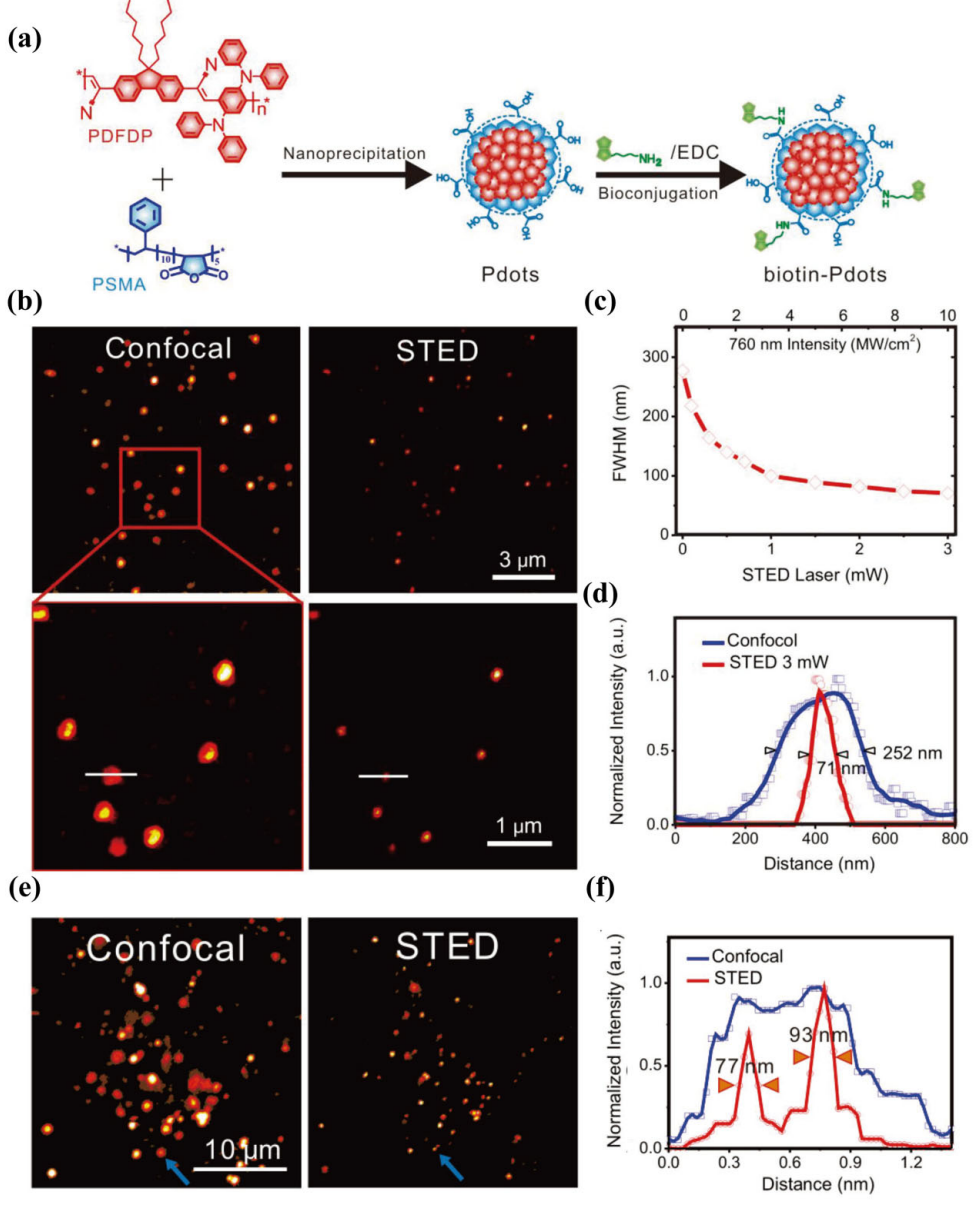
Super-resolution imaging of PDs. (**a**) Schematic illustration of synthetic route of biotin–PDs. (**b**) Images of PDs captured by Confocal and STED microscope. The same PDs were used for the confocal and STED imaging with and without the 760 nm depletion beam, respectively. The lower panel is a confocal and STED images of the enlarged red box area from in the top panel. (**c**) Correlation of single-particle FWHM with the laser power of the STED beam changing from 0 to 3 mW (0 to 10 mW/cm^2^). (**d**) Normalized intensity profiles of the single PDs along corresponding white lines in (**b**). (**e**) Confocal and STED images of a HeLa cell incubated with biotin–PDs, suggesting that biotin–PDs were uptaken by the cell. (**f**) Normalized intensity profiles of the PDs denoted by the blue arrows in (**e**). Adapted with permission from [66].

The Fang group pioneered the use of PDs for STED imaging, which involves using poly[{9,9-dihexyl-2,7-bis(1-cyanovinylene) fluorene}-alt-co-{2,5-bis(N,N0-diphenylamino)-1,4-phenylene}] (PDFDP) PDs with an absorption peak at 395 nm and emission peak at 668 nm as the fluorescent probe [66]. As shown in Figure 3, using a 760 nm depletion beam, the PDFDP PDs can achieve a resolution of 71 nm at the single-particle level. Furthermore, biotinylated PDFDP PDs, which are utilized as specific markers for receptor-mediated endocytic vesicles, exhibit a resolution of 77 nm in vivo and allow for the visualization of the dynamic fusion and fission of vesicles within live cells, enabling continuous STED imaging for up to 33 min. In another work, the Fang group further investigated the interaction of cellular organelles and vesicles in living cells with bright and photostable PDs [67]. Specifically, they used a pair of PDs, poly[2-methoxy-5-(2-ethylhexyloxy)-1,4-(1-cyanovinylene-1,4-pheny-lene)] PDs and PDFDP PDs, to collect signals ranging from 560 to 600 nm and 660 to 700 nm for dual-color STED imaging, excited solely by a 506 nm beam and depleted by a 760 nm beam. The simultaneous labeling of membrane protein CD44 and microtubule filaments was performed using these PDs and the dynamic interactions among endosomes were detected and tracked for 30 min, underscoring the potential of using PDs for long-term STED imaging.

While the PDs have shown a promising performance in STED, challenges remain. Despite the fact that PDs are highly photostable in general, there are PDs with photo-blinking behavior, and some polymers are less resistant to photo-oxidation, which should be considered in STED imaging. In addition, while there reports of small PDs of a few nanometers, most of the PDs seem to be in the size range of tens of nanometers and the size distribution is relatively heterogeneous compared to QDs, which makes it difficult to control the surface-functional groups and biomolecules attached to the PDs surface. As a result, the high-density, uniform, continuous labeling of biostructures could be challenging, depending on the PDs used for labeling.

### 2.3. CDs

**Figure 4:**
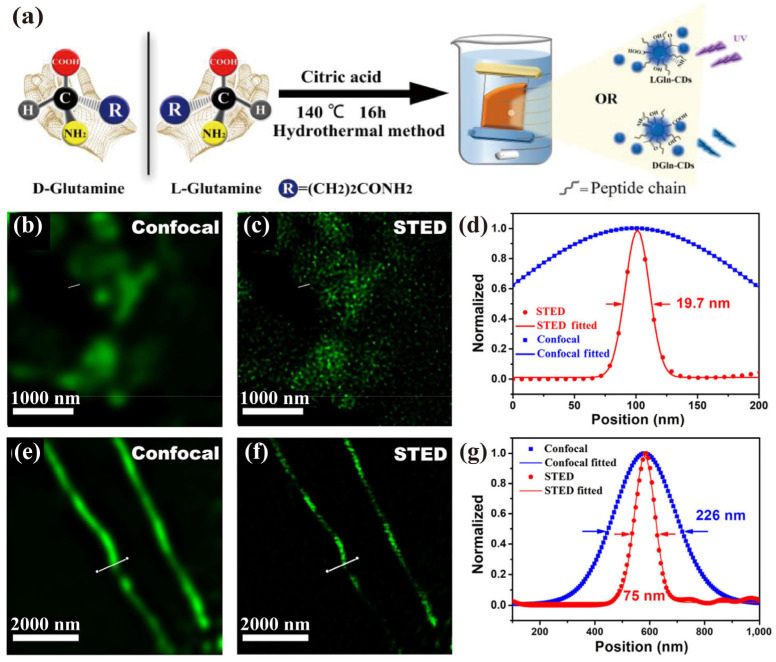
(**a**) Illustration of representative synthetic route of CDs. Adapted with permission from [69]. (**b**) The magnified confocal and (**c**) corresponding STED microscopy image of the nucleus of the cell. (**d**) Intensity profiles along lines shown in (**b**,**c**), respectively, across the CDs in the nucleus of the cell. The fluorescence intensity was normalized. (**e**) The enlarged confocal microscopy and (**f**) corresponding STED microscopy image of tunneling nanotubes. (**g**) Intensity profiles along lines demonstrated in (**e**,**f**) across the tunneling nanotubes. Fluorescence intensity was normalized. The 470 nm excitation laser and 775 nm depletion laser were fixed in 1.0 and 39.6 mW, respectively. Adapted with permission from [73].

CDs, a zero-dimensional carbon nanomaterial, are fluorescent particles with a size below 10 nanometers (Figure 4a) [68,69,70]. The luminescent mechanism of CDs primarily arises from quantum confinement and surface defect effects. Their nanoscale structure confines electrons in all three dimensions, leading to discrete energy levels analogous to QDs. Upon optical excitation, electrons transition from the valence band to the conduction band, releasing energy as fluorescence. The surface defects on CDs act as luminescent centers, modulating fluorescence wavelength and intensity. Although CDs and QDs share a similar luminescent mechanism based on quantum confinement, they differ significantly in their composition and properties. CDs are primarily composed of carbon, exhibiting excellent water solubility, low toxicity, and environmental friendliness. In contrast, traditional QDs may pose environmental and biological hazards due to their heavy metal content. Furthermore, the luminescent properties of CDs can be finetuned by manipulating their size and surface states, while QDs’ luminescence strongly depends on their composition and crystal structure. In addition to these advantages, CDs exhibit a remarkable fluorescence quantum yield (QY), photostability, ease of functionalization, and cost-effectiveness, making them promising candidates for the super-resolution imaging of biological structures [71].

Pompa et al. pioneered the use of CDs (5.5 ± 0.7 nm) with a peak emission at 490 nm for subcellular imaging in STED microscopy [72]. By dispersing the CDs on a coverslip and exciting them at 405 nm with depletion at 592 nm, they demonstrated evident super-resolution. The confocal and STED imaging revealed FWHM values of 170 nm and 54 nm, respectively, representing a 3.1-fold improvement in lateral resolution. Additionally, they studied the uptake of CD by MCF-7 cells and found that they can be enriched in lysosome via colocalization with a lysotracker dye. STED imaging achieved resolutions of 30 nm for fixed and 67 nm for live MCF-7 cells. Qu et al. further enhanced the optical properties of CDs by doping with N and F, introducing more defect sites [73]. As shown in Figure 4b–d, they used these doped CDs to label and image the nucleolus and tunneling nanotubes, achieving resolutions of 19.7 nm and 75 nm, respectively. The Zhang group reported the development of 2–3 nm CDs capable of emitting distinct colors upon binding to double-stranded DNA and single-stranded RNA. Owing to their nanoscopic dimensions, these CDs are able to traverse the cellular membrane barrier, thereby facilitating the real-time visualization of DNA and RNA localization and dynamics within live cells using STED imaging [74]. Additionally, the Wu group demonstrated nucleus-targeted STED imaging using red-emitting CDs synthesized with metal ions as catalysts during the hydrothermal treatment of p-phenylenediamine. The synthesized Ni-pPCDs demonstrated a QY of 45.6%, good photostability, and selective accumulation in the nucleoli. Following their administration in living cells, a resolution of 80 nm was achieved through wash-free STED imaging utilizing an excitation wavelength of 552 nm and a depletion wavelength of 660 nm [75]. In another case, the Wu group developed CDs with bacterial differentiation capabilities. The fluorescence emission of the CDs is polarity-sensitive, resulting in significantly enhanced fluorescence when the CDs interact with bacteria. The presence of both hydrocarbon chains and positively charged quaternary ammonium groups lead to the selective targeting of Gram-positive bacteria. Under the STED imaging mode, small aggregates of CDs can be clearly observed on the bacteria membrane, showing that STED microscopy can be used to study interactions between the CDs and bacteria [76].

Despite their promising performance, several limitations hinder the widespread application of CDs in STED imaging. Firstly, the development of red-emitting CDs remains a crucial unmet need. Secondly, CDs also exhibit broad excitation and emission spectra, posing a challenge to multicolor imaging with CDs. Addressing these limitations is essential for the further advancement of CD-based STED imaging for biological research.

### 2.4. UCNPs

Upconversion luminescence refers to the process of absorbing two or more low-energy photons to emit one high-energy photon, which is a nonlinear optical process with an anti-Stokes shift. The upconversion process in rare earth elements mostly occurs during transitions between 4f electronic configurations. The electron absorbs photon energy to jump to an excited state, and then acquires the energy of another photon to undergo another upward transition to reach a higher-energy excited state level. From this level, the electrons undergo radiative transitions back to the ground state, completing the upconversion process [77,78,79]. UCNPs, i.e., FNPs doped with rare earth ions [80,81] demonstrate luminescence through the anti-Stokes shift, which is distinctively different from conventional fluorophores, providing several advantages including a deeper penetration depth and lower background auto-fluorescence. These properties have positioned UCNPs as a promising class of fluorescent probes for STED imaging [82,83].

**Figure 5:**
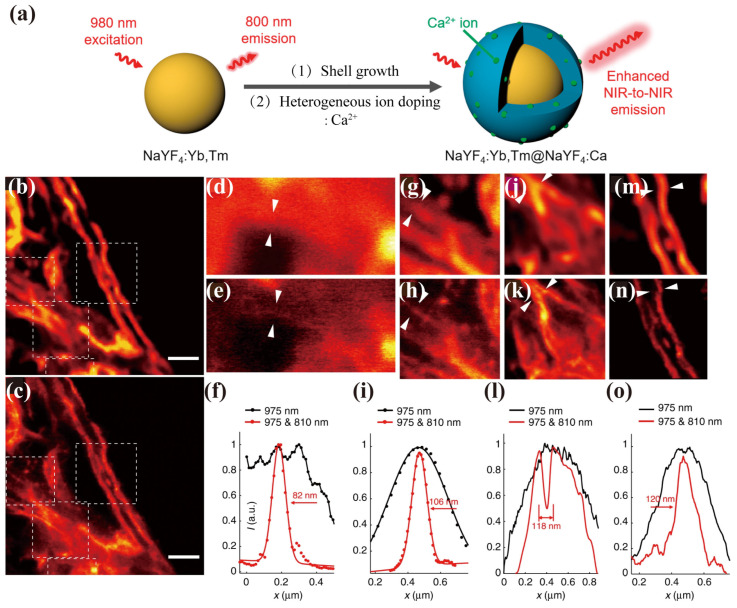
(**a**) Illustration of the representative synthetic route of core/shell UCNPs. Adapted with permission from [78]. (**b**) The multiphoton microscopy image and (**c**) corresponding image of the same region obtained in the super-resolution mode of UCNPs labelled cytoskeleton and desmin proteins in HeLa cells (975 nm excitation and the 810 nm STED laser beam). Scale bars: 2 μm. (**d**–**o**) magnified images of selected areas from (**b**,**c**) (indicated by white dotted squares) and corresponding line profile analysis. White dotted squares in (**b**) are shown in (**d**,**g**,**j**,**m**). White dotted squares in (**c**) are shown in (**e**,**h**,**k**,**n**). (**f**,**i**,**l**,**o**) Line profiles’ analysis of region of interest, as indicated by arrow heads in (**d**,**e**,**g**,**h**,**j**,**k**,**m**,**n**). Adapted with permission from [84].

The He group introduced an efficient optical depletion method leveraging interionic cross-relaxation, significantly reducing the depletion beam intensity [84]. Using a multiphoton laser scanning microscope, they demonstrated this approach using NaYF4:18% Yb^3+^, 10% Tm^3+^ (18.0 ± 1.8 nm) UCNPs. Imaging with a 975 nm excitation beam and 810 nm depletion (17.7 mW/cm^2^) beam achieved a lateral resolution of 66 nm. Impressively, the scanned area retained the imaging quality even after 3000 scans, without photobleaching or photo-blinking. They further applied this method to image cellular cytoskeleton protein desmin using antibody-conjugated UCNPs, achieving a lateral resolution of 82 nm (Figure 5b–o). Meanwhile, the Liu group also demonstrated STED imaging with a reduced excitation and depletion intensity using UCNPs doped with 8% Tm^3+^ ions and 20% Yb^3+^, achieving a 28 nm resolution when excited at a 980 nm wavelength with 0.66 mW power and depleted at an 808 nm wavelength with 7.5 mW cm^−2^ power [54]. In this way, a lower saturation of 0.19 mW cm^−2^ intensity was achieved with STED microscopy. Additionally, the Jin group recently reported on the utilization of the unique upconversion emission responses from UCNPs in super-resolution microscopy, achieving super-resolution using a single doughnut-shaped scanning excitation beam, without the need for a depletion beam. By capturing the four-photon emission from individual UCNPs, high-frequency details of super-resolution images can be resolved accurately through the doughnut-emission PSF. Concurrently, by over-saturating the two-photon states within the same nanoparticle, complementary low-frequency information was obtained from a Gaussian-like emission PSF. This approach enabled the development of a Fourier domain heterodyne fusion method, enabling the engineering of PSF to capture both low- and high-frequency details, ultimately leading to optimized image quality. Impressively, they achieved a spatial resolution of 40 nm, representing a remarkable improvement of 1/24th of the excitation wavelength, offering a novel method for the use of nonlinear multicolor emission probes for super-resolution imaging [85]. Next, the same group also developed a rate equation model to optimize the excitation intensity, balancing the depletion efficiency and the imaging quality. The authors demonstrated that the resolution of Tm^3+^-doped UCNPs can easily achieve sub-40 nm resolution using the STED imaging model (~1.0 mW cm^−2^ excitation, 3.4 mW cm^−2^ depletion), which is 1/24th of the excitation wavelengths employed. These findings provide opportunities for population control in the realization of low-power, high-resolution nanoscopy [86].

However, despite their unique properties, UCNPs still face several challenges that hinder their widespread utilization. One of the most significant issues is their relatively low-luminescence QYs, which typically remain below 1%. Additionally, due to the presence of heavy metal, there are biological safety concerns that need to be addressed. The development of new materials and synthetic conditions is required to address these challenges and advance their applications in STED microscopy.

### 2.5. AIEs

The AIE materials exhibit weak fluorescence in solution but intense fluorescence in aggregated states, contrary to the Aggregation-Caused Quenching (ACQ) effect commonly observed for conventional fluorophores. The AIE effect is related to the intramolecular motion of the fluorophore. In solution, the AIE fluorophores exhibit a strong intramolecular rotation, which quickly dissipates the absorbed photon energy and results in weak fluorescence emissions, while in the aggregated state, the intramolecular rotation is restricted by the spatial hindrance, which leads to enhanced-fluorescence QY. AIEs consist of AIE fluorophores encapsulated in a nanoparticle, offering advantages such as low toxicity, a large Stokes shift, high fluorescence efficiency in the aggregation state, and strong photostability [87,88], making them a promising class of FNPs for STED imaging.

**Figure 6:**
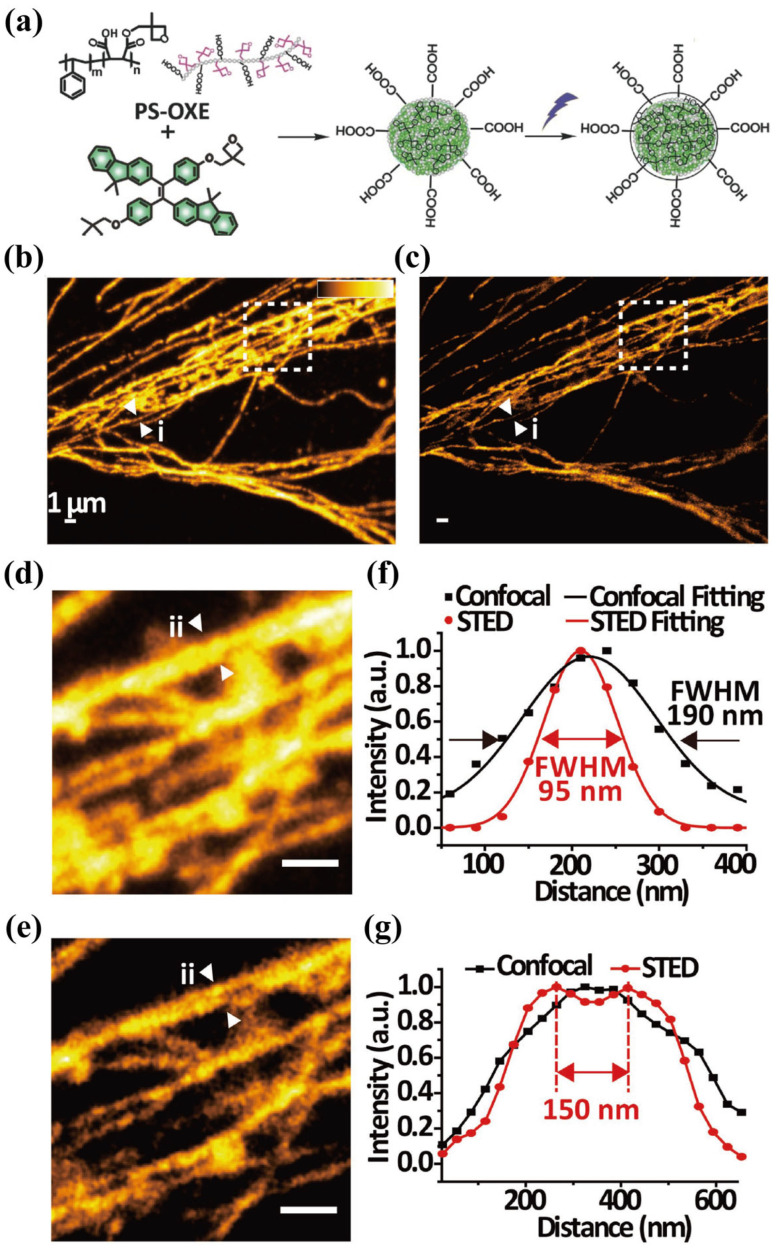
(**a**) Schematic illustration for the preparation of small AIEs by photo-crosslinking. (**b**) Confocal microscopy and (**c**) STED microscopy images of the microtubules. (**d**,**e**) Enlarged views of the region of interest, denoted by dotted squares in (**b**,**c**). (**f**) Line profiles of the interest position demonstrated by arrowheads (i) in (**d**,**e**). (**g**) Line profile of the position of interest shown by the arrowheads (ii) in (**d**,**e**). The scale bar in (**b**–**e**) represents 1 μm. Adapted with permission from [89].

Sun and Wu’s group synthesized blue-, green-, and red-emitting AIEs using nanoprecipitation and photo-crosslinking [89]. These dots were conjugated with streptavidin for the specific labeling of membrane epithelial cell-adhesion molecule and microtubules via antigen–antibody recognition. As shown in Figure 6, the confocal and STED imaging of microtubules labeled with Red-AIEs revealed an FWHM of 95 nm, showing finer microtubule structures compared to the 190 nm FWHM in confocal mode. Although three AIEs were reported, multi-color labeling and STED imaging were limited due to spectral crossover and potential re-excitation by the STED beam. Subsequently, the Meng group synthesized red AIEs with an emission range of 550–850 nm, prepared via a facile nanoprecipitation method. The AIEs were designed with a donor–acceptor structure centered on DTPA-BT-F as the molecular backbone, aiming to enhance non-covalent intermolecular interactions. The resulting AIEs exhibited a particle size of 50 nm, a Stokes’ shift of 175 nm, and a photoluminescence (PL) QY of 36.49%. By labeling lysosomes, the group achieved a resolution of 107 nm in STED mode versus 548 nm in confocal mode, highlighting the potential of these probes in live cell STED imaging applications [90]. Recently, the Tang group reported solvatochromic near-infrared AIEs exhibiting a QY of 98.4% in low-polarity solvents and 26.8% in the solid state [91]. Facilitated by the low saturation depletion power of 0.83 mW/cm², these dots enable the high-resolution STED imaging of lipid droplets, achieving a resolution of 62 nm. In another work, the Qian and Tang groups doped the typical AIE dye 2,3-Bis(4-(phenyl(4-(1,2,2-triphenylvinyl)phenyl)amino)phenyl) fumaronitrile into mesoporous silica, resulting in FNPs with a diameter of 24.3 nm. The FNPs exhibited an absorption peak at 510 nm and a fluorescence peak at 660 nm, demonstrating a Stokes shift of 150 nm. When employed in STED imaging, excitation was performed at 594 nm, depletion occurred at 775 nm, and the emissions were collected in the range of 650–720 nm. Single-particle STED imaging achieved a lateral resolution of 30.7 nm, an eightfold improvement compared to confocal microscopy, which closely approximated the actual size of the nanoparticle [92].

In summary, the AIEs exhibit remarkable promise for applications in STED microscopy. Nevertheless, the precise control of particle size and surface-functional groups remains a challenge. Furthermore, the relatively broad excitation and emission bandwidths pose limitations on the development of multicolor imaging using AIEs. To address these challenges, further research is needed to develop new synthetic strategies and surface modification techniques. Additionally, the exploration of new AIE dyes with narrower excitation and emission bandwidths could pave the way for the development of multicolor STED microscopy.

### 2.6. MCs

Atomically precise MCs with sizes typically below 2 nm bridge the gap between discrete atoms and plasmonic nanomaterials [93,94,95]. These clusters exhibit quantum size effects and discrete electronic states, leading to unique optical properties. Their precise atomic composition confers molecule-like optical characteristics distinct from those of plasmonic NPs, which are typically in the size range of tens of nanometers. This atomic precision enables a profound understanding of structure–property relationships, facilitating the design of MCs tailored for specific applications. Furthermore, their good biocompatibility and reduced toxicity highlight their potential as imaging agents. Their exceptionally small size compared to other FNPs enhances labeling density and imaging resolution. This allows for precise monitoring of the dynamics and distribution of biomolecules with minimal perturbations.

**Figure 7:**
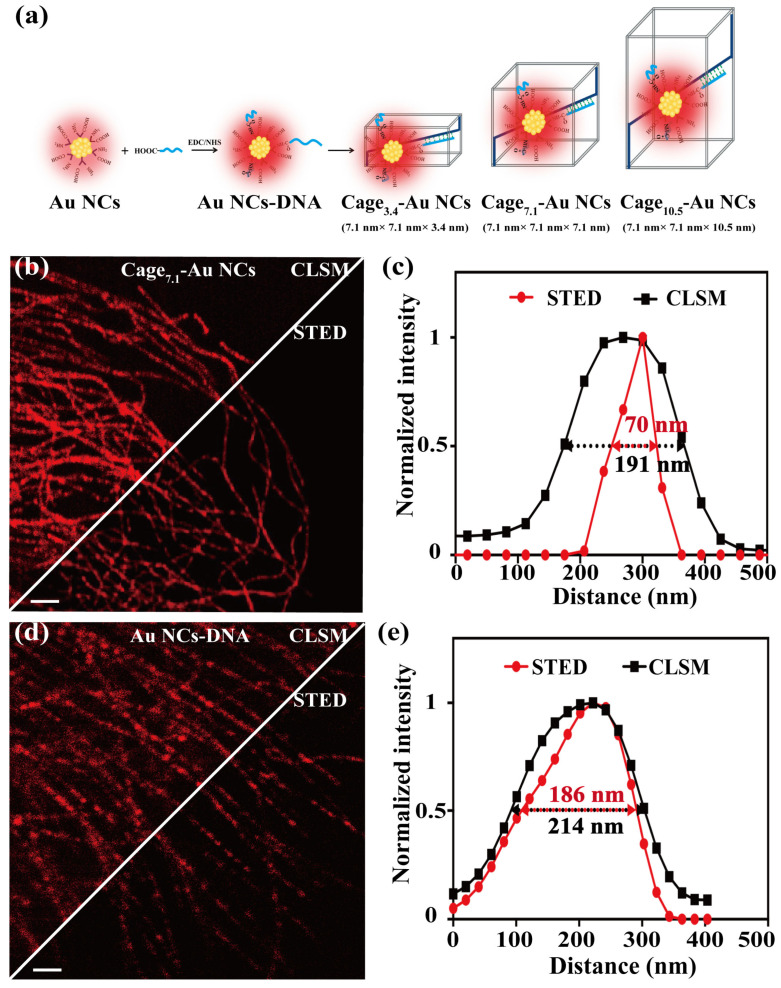
(**a**) Schematic illustration of the encapsulation of DNA-functionalized Au NCs into a DNA cage with different sizes. (**b**) The confocal (upper) and STED (lower) images of antibody-conjugated Cage_7.1_-Au NCs labelled microtubules. Scale bar is 5 μm. (**c**) Representative normalized intensity profiles of the Cage_7.1_-Au NCs. (**d**) Confocal microscopy (upper) and STED microscopy (lower) images of the antibody-conjugated Au NCs-DNA labelled microtubules. Scale bar is 5 μm. (**e**) Representative normalized intensity profiles of the Au NCs-DNA. Adapted with permission from [97].

As first demonstrated by the Fang group, ultrasmall and ultrabright gold nanoclusters (Au NCs), fabricated using a rigid shell template of L-arginine and 6-aza-2-thiothymine ligand [96], have been successfully applied as STED imaging probes. These Au NCs, averaging 1.7 nm in size, emit green light and achieve a remarkable STED imaging resolution of 24 nm, which is among the best in FNPs. However, Au NCs show polydispersity and environmental sensitivity, and lead to challenges in controlling surface functional group stoichiometry. In order to overcome these limitations, the same group developed a novel method using DNA cages (7.1 × 7.1 × 7.1 nm^3^) to encapsulate ultrasmall Au NCs. This approach results in monodispersed and monofunctional Au NCs, which exhibit significantly enhanced fluorescence brightness, photostability, and environmental resistance. As shown in Figure 7, imaging with STED microscopy achieved a resolution of ~40 nm at the single-particle level. By specifically labeling the nucleus with AS1411 aptamer at a 1:1 stoichiometric ratio, the DNA-caged Au NCs demonstrated a superior imaging contrast and better photostability compared to free Au NCs. The immunostaining of microtubules further revealed the superior labeling density and homogeneity of DNA-caged Au NCs [97]. Wang’s group developed Ag clusters functionalized with the AS1411 aptamer, targeting the nucleolin protein in cancer cells [98]. The AS1411-conjugated Ag NCs emit at 640 nm and exhibit a fluorescence QY of 35% and high photostability with minimal blinking during imaging. In the STED imaging mode, these clusters demonstrate a clear narrowing effect with a resolution of 55 nm, enabling a distinction between closely located clusters compared to confocal microscopy. Upon incubation with HeLa cells and subsequent confocal and STED imaging, it was observed that the AS1411-conjugated Ag NCs exhibited remarkable specificity for nucleolin protein labeling within the cytoplasm, providing vastly improved contrast and resolution in the imaging of the target.

As shown above, researchers have performed detailed investigations of the structure–properties relationships of MCs, which allows for precise control of the MCs’ optical properties for STED imaging. However, MCs are sensitive to the surrounding environment and their fluorescence properties can be influenced by factors such as pH and ion concentration. Furthermore, aggregation or precipitation may occur under certain conditions, especially in complex physiological environments. The functionalization of metal nanoclusters enhances their utility in imaging. However, surface modifications can affect the original properties, potentially altering their performance and necessitating careful evaluation for specific applications.

### 2.7. NDs

**Figure 8:**
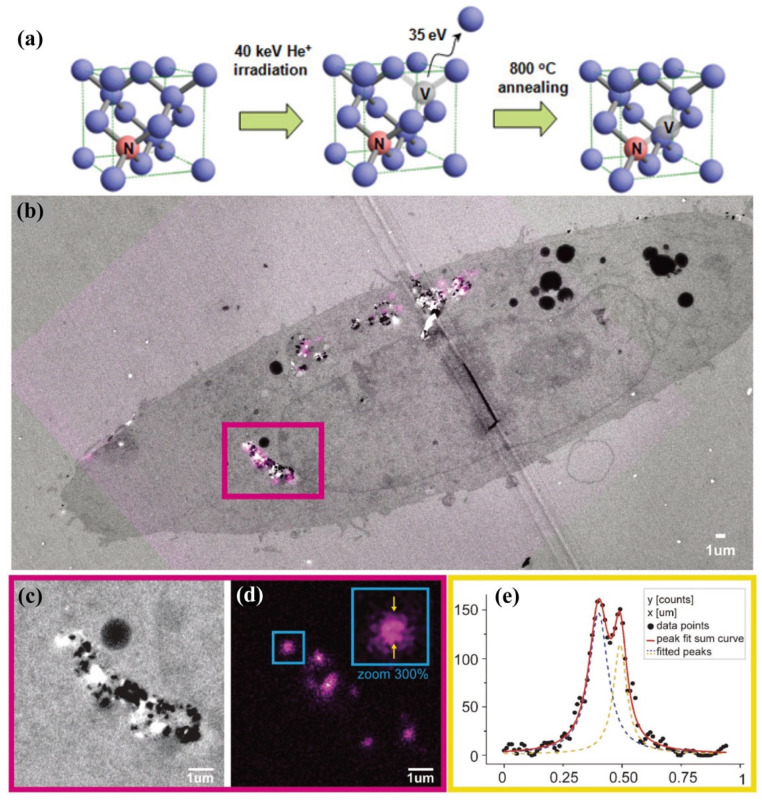
(**a**) The illustration of the representative preparation of the NV centers in type-Ib diamond nanocrystallites. Adapted with permission from [99]. (**b**) Image of a single cell merged with TEM images, shown by gray, and fluorescence images, shown by magenta. Enlarged views of the pink square in the merged image indicating (**c**) TEM and (**d**) STED images, respectively. For the blue box, the right is the 3x magnification of the left. (**e**) Line profile along the arrow in (**d**) and corresponding Lorentzian fit. The two distinct peaks are ~90 nm apart. Adapted with permission from [102].

The fluorescence emission mechanism of NDs is related to the presence of impurity elements, particularly nitrogen (Figure 8a) [99]. When exposed to ultraviolet (UV) light, nitrogen impurities within the nanodiamonds absorb UV photons, leading to the excitation of electrons, which simultaneously relax back to the ground state and emit fluorescence photons. Fluorescent NDs possess unique characteristics, including a small size, tunable PL emission, excellent biocompatibility, and exceptional photostability, rendering them suitable for use as probes in STED microscopy [100,101]. Their photostability is attributed to the stable and chemical inert crystal structure of NDs. The fluorescence color that is emitted is specific to the NDs’ size, impurity element species, concentration, and state. By synthesizing NDs with different fluorescent colors, multicolor imaging can be achieved, enabling the simultaneous monitoring of multiple biological molecules and cellular processes. The remarkable photostability of NDs facilitates prolonged observations, which are crucial for capturing dynamic biological events.

In 2009, the Hell group first used STED microscopy to image NV centers in NDs beyond the diffraction limit [102]. They mapped NV centers in a series of red NDs ranging from 40 to 250 nm in size [52]. Specifically, when resolving single NVs in individual NDs, they obtained a FWHM of 9.5 nm, compared to the 325.8 nm obtained in confocal imaging, representing a 34-fold increase in resolution. Multiple NVs in a single ND could be clearly resolved with a resolution as high as 10 nm. This work confirmed that NDs with nitrogen impurities can be used in STED microscopy. The Laporte group innovatively developed green-emitting NDs STED probes by incorporating Nitrogen–Vacancy–Nitrogen centers and achieved a resolution of 70 nm in STED imaging, enriching the fluorescence emission colors and providing opportunities for multicolor STED imaging using NDs [103]. Due to their excellent optical properties and dense electron nature, the Rosenholm group utilized NDs for STED-TEM correlative microscopy [104]. MDA-MB-231 cells stained with NDs were processed with fixatives and heavy metal staining to meet the requirements for TEM, which did not compromise the fluorescent properties of the NDs. As shown in Figure 8b–e, the NDs exhibited excellent contrast in TEM imaging. Furthermore, a resolution of 90 nm was achieved in STED imaging, demonstrating the feasibility of using NDs in both TEM and STED imaging modes.

Despite the exceptional advantages of NDs in long-term STED imaging, their further development remains constrained by several challenges. Notably, their intricate and demanding preparation process poses a significant hurdle, limiting their widespread utilization. Additionally, the limited emission wavelength range restricts their use in multicolor imaging. Particle aggregation also complicates their use in biological settings. Addressing these limitations is crucial for the continued development of fluorescent NDs and for realizing their full potential in STED imaging.

### 2.8. Dye-Loaded NPs

A straightforward way to enhance the brightness and photostability of fluorescent probes in imaging is to pack multiple organic dyes into a nanoparticle. However, organic dyes are often suspected to undergo ACQ in the aggregated state. Strategies such as using mesoporous substrate, introducing spatial hindrance units, or chemical cross-linking, are typically involved in the preparation of dye-loaded NPs. One of the most well-known examples of these materials are dye-loaded silica NPs, which have been widely used in applications such fluorescence imaging, bio-sensing, and light-emitting devices [105]. STED imaging using dye-loaded NPs, however, is still an emerging area. Since STED microscopy requires the sensitive depletion response of the fluorescent probe in addition to high brightness and good photostability, additional consideration is required in the development of dye-loaded NPs for STED imaging.

**Figure 9:**
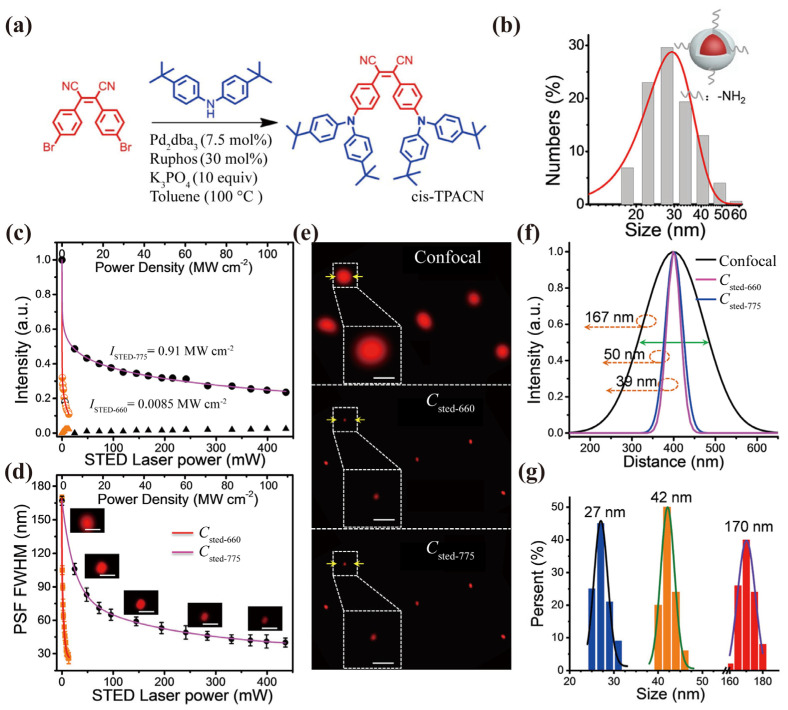
(**a**) Illustration of synthetic route of the TPACN dimer. (**b**) Size distribution of TPACN-loaded NPs characterized by dynamic light scattering. (**c**) Efficiency of stimulated emission depletion and fluorescence background resulted from Sthe TED beam irradiation of TPACN-loaded NPs. The percentages of fluorescence intensity of TPACN-loaded NPs under an irradiation of 470 nm excitation and 660 nm (orange dots) or 775 nm (black dots) depletion at different depletion powers. (**d**) Correlation of PSF FWHM and STED laser power obtained at 660 nm or 775 nm depletion light for TPACN-loaded NPs with increased power. Several representative single NPs STED images are also shown with 775 nm depletion light. (**e**) The confocal microscopy image of TPACN-loaded NPs excited at 470 nm (0.05 KW cm^−2^) and corresponding STED microscopy image with an additional depletion beam of 660 nm (3.31 mW cm^−2^) or 775 nm (109 mW cm^−2^). For the white dashed box, the lower is the magnified version of the corresponding figure above. Scale bar: 100 nm. (**f**) Fluorescence intensity profiles of single TPACN-loaded NPs, indicated by the yellow arrow in (**e**). (**g**) The observed size distribution of TPACN-loaded NPs under confocal (0.05 KW cm^−2^, red histogram), additional 660 nm depletion (3.31 mW cm^−2^, blue histogram), and additional 660 nm depletion (109 mW cm^−2^, orange histogram). The dwell time of each pixel was 12 μs. 1024 pixels × 1024 pixels. Scale bar in (**b**) is 200 nm and that in the magnified image in (**b**,**c**) is 100 nm. Adapted with permission from [105].

The Shang and Nienhaus group developed dye-loaded NPs by cross-linking dyes with proteins. They utilized a click-reaction to conjugate Atto647N-NHS-ester with transferrin (Tf), which can target cancer cells with Tf receptors, followed by a crosslinking reaction using glutaraldehyde and purification steps. The resulting Tf-based dye-loaded NPs exhibited a diameter of 25 nm and exhibited a slight blue shift in the emission maximum compared to free atto-647 dye. These dye-loaded NPs demonstrated significantly enhanced photostability, biocompatibility, and specific cellular targeting. STED imaging showed a superior resolution compared to confocal imaging, with dye-loaded NPs being resolved at 64 nm versus 242 nm at the single-particle level [106]. The Fu group constructed four sub-5 nm dye-loaded NPs, utilizing the host–guest interaction between isopercolic acid derivatives and cyclodextrin. These dye-loaded NPs exhibited a dramatically improved fluorescence quantum yield (FL QYs before and after incorporation: 45.6% vs. 12.8%; 38.9% vs. 16.4%; 18.8% vs. 0.3%; 15.2% vs. 0.4%), excellent photostability, and a low saturation intensity of 0.5 mW/cm^2^. STED imaging achieved a resolution of 30 nm, indicating their great potential as probes for STED imaging [107]. Moreover, silica, which is often utilized to encapsulate dyes for further functionalization, was employed by the Fu group to encapsulate TPACN dimers into a quasi-spherical geometry to prevent ACQ [105,108]. The resulting dye-loaded NPs are 28 nm in size and the maxima absorption and emission are ~500 nm and ~650 nm, respectively, showing the solvent dependence. These NPs, if utilized in STED imaging, can be effectively depleted by both 660 nm and 775 nm lasers, without causing re-excitation upon the introduction of the 660/775 nm depletion beam. When depleted at 660 nm, a single-particle resolution of 25 nm is achieved, with a minimal saturation intensity of 0.0085 mW cm^−2^. In comparison, at 775 nm depletion, a single-particle resolution of 40 nm is obtained, with a minimal saturation intensity of 0.91 mW cm^−2^ (Figure 9). Additionally, the dye-loaded NPs exhibit remarkable photostability, with their fluorescence signal remaining nearly unchanged after 30 s of simultaneous exposure to both excitation and depletion light. Utilizing STED imaging, the long-term dynamics of lysosomes within live cells were analyzed. At a resolution of 34 nm, dynamic and disordered lysosome movements were observed. Furthermore, the intricate processes of lysosome fusion and fission were successfully visualized. High-efficiency deep-red emissions are highly desirable for in vivo imaging. Recently, dye-loaded NPs based on donor-acceptor-type DBTBT-4C8 bearing flexible alkyl chains was developed, showing tunable deep-red emissions from 600 to 800 nm, with QYs as high as 25%. The STED imaging of fixed HeLa cells and glass cat fishes was demonstrated and achieved a resolution of ~100 nm, showing the potential of using these dye-loaded NPs for both in vitro and in vivo imaging applications [109].

However, despite these advances, the application of dye-loaded NPs as probes in STED imaging also encounters several challenges. The multi-step synthesis process requires careful control over the reaction conditions, thereby increasing the overall complexity of the preparation. Some dye-loaded silica NPs are prone to aggregation and degradation, which can result in low labelling homogeneity and relatively low stability in STED imaging. Furthermore, the relatively large size of dye-loaded NPs can lead to a low labelling density, limiting their capacity to capture subcellular details. Overall, further development of the synthesis and functionalization methods of dye-loaded NPs is required in order to address these challenges and advance their applications in STED imaging.

## 3. Conclusions and Outlook

Overall, STED, as a powerful bio-imaging technique, has revolutionized our understanding of cellular structures and processes below the diffraction limit. As compared to conventional fluorescent probes like organic dyes and fluorescent proteins, FNPs present several advantages for STED imaging. This comprehensive review explores the latest advances in FNPs for STED microscopy, culminating in the following pivotal conclusions. Firstly, their exceptional brightness and sensitive depletion response allow for enhanced signal-to-noise ratios, crucial for achieving high-resolution STED imaging. Furthermore, FNPs typically demonstrate remarkable photostability, enduring prolonged exposure to intense laser light without significant photobleaching, thereby ensuring prolonged imaging sessions and improved data quality. Their tunable optical properties, including their absorption/emission wavelength and QY, can be precisely controlled, providing researchers with versatile tools optimized for specific experimental conditions and biological samples. Additionally, the ability to control the size of FNPs enables researchers to match probe dimensions with the resolution requirements of their investigations, further enhancing imaging precision.

However, biological safety is a crucial factor limiting the application of FNPs in the field of biomedicine. Different types of FNPs possess distinct biological safety characteristics and potential risks. Inorganic FNPs, such as QDs and UCNPs, may pose a greater risk due to the presence of heavy metals. In comparison, organic FNPs, including PDs, AIEs, CDs, NDs, and dye-doped NPs, have lower safety risks, making them more suitable for live-cell and in vivo imaging applications. Nevertheless, a detailed biological safety assessment encompassing the behavior in biological systems and metabolic pathways, the long-term safety, and the impact on human health and the environment is still required. Additionally, the development of more effective biological safety assessment methods and standards is necessary to provide scientific evidence for the safe application of fluorescent nanomaterials.

Looking ahead, future directions for leveraging FNPs in STED imaging encompass advancements in resolution through innovative nanoparticle designs, the refinement of functionalization strategies for targeted labeling, the exploration of in vivo imaging possibilities for studying complex biological phenomena, finding FNPs with better biosafety, integration with complementary imaging modalities for comprehensive analysis, and efforts to promote commercialization and accessibility, democratizing access to this powerful microscopy technique and fostering new discoveries in biology and medicine. These endeavors hold the potential to propel STED microscopy to even greater heights, enabling researchers to unravel the intricacies of cellular dynamics with unprecedented clarity and detail.

## Figures and Tables

**Table 1:** The advantages and disadvantages of the various FNPs in STED microscopy.

Type	Advantages	Disadvantages
QDs	Photostable; Narrow emission band; High PL QY.	Broad absorption band and risk of multiphoton excitation; Potential toxicity of heavy metals; interference from photo-blinking.
PDs	Large extinction coefficients; Easy functionalization; Good photostability; High biocompatibility; Fast fluorescence radiation rate.	Difficult to control particle size and surface functional groups.
CDs	Facile preparation; Superior biocompatibility; Easy surface functionalization.	Lack red and infrared emission; Broad absorption bandwidths.
UCNPs	Sharp emission band; Deep tissue penetration; Avoids background autofluorescence; Photostable.	Poor water solubility; Low PL QY; Excitation/emission bands are nearly invariable; Potential photothermal effect; Potential toxicity of metals; Large size.
AIEs	Tunable particle size; Easy surface functionalization; Avoid aggregation-induced quenching through AIE effect.	Difficult to control particle size and surface functional groups; Relatively broad excitation and emission bandwidths.
NDs	Biocompatible; High PL QY; Remarkable photostability.	Difficult to prepare; Limited emission wavelength; Tendency to aggregate.
MCs	Small size; Tunable PL emission; Photostable.	Low PL QY; Tendency to aggregate; Sensitive to environment.
Dye-loaded NPs	Biocompatible; Broad choice of fluorophores.	Prone to aggregation-induced quenching; Difficult to control dye-loading efficiency.

## References

[1] Sun N., Jia Y., Bai S., Li Q., Dai L., Li J. (2023). The power of super-resolution microscopy in modern biomedical science. Adv. Colloid Interface Sci..

[2] Grove J. (2014). Super-resolution microscopy: A virus’ eye view of the cell. Viruses.

[3] Kim M.J., Park H.J., Jung R.J., Won C.Y., Ohk S.O., Kim H.T., Roh N.K., Yi K.H. (2024). High-resolution 3-D scanning electron microscopy (SEM) images of DOT(TM) polynucleotides (PN): Unique scaffold characteristics and potential applications in biomedicine. Skin Res. Technol..

[4] Mentor S., Cummings F., Fisher D. (2022). Preparation of biological monolayers for producing high-resolution scanning electron micrographs. PLoS ONE.

[5] Ede J.M., Beanland R. (2020). Partial Scanning Transmission Electron Microscopy with Deep Learning. Sci. Rep..

[6] Wang S., Cheng Y., Liu L., Chen R., Li Y., Wang H., Zhang R. (2024). The Morphology and Ultrastructure of Dermal Telocytes Characterized by TEM and AFM. Cell Biochem. Biophys..

[7] Tizro P., Choi C., Khanlou N. (2019). Sample Preparation for Transmission Electron Microscopy. Methods Mol. Biol..

[8] Xie L., Hong J. (2022). Transmission Electron Microscopic Methods for Plant Virology. Methods Mol. Biol..

[9] Wang Y., Sun B., Shibata B., Guo F. (2022). Transmission electron microscopic analysis of myelination in the murine central nervous system. STAR Protoc..

[10] Elliott A.D. (2020). Confocal Microscopy: Principles and Modern Practices. Curr. Protoc. Cytom..

[11] Pack C.G. (2021). Confocal Laser Scanning Microscopy and Fluorescence Correlation Methods for the Evaluation of Molecular Interactions. Advanced Imaging and Bio Techniques for Convergence Science.

[12] Ulrich M. (2015). Konfokale Laserscanmikroskopie [Confocal laser scanning microscopy]. Hautarzt.

[13] Fish K.N. (2022). Total Internal Reflection Fluorescence (TIRF) Microscopy. Curr. Protoc..

[14] Midorikawa M. (2018). Real-time imaging of synaptic vesicle exocytosis by total internal reflection fluorescence (TIRF) microscopy. Neurosci. Res..

[15] Mattheyses A.L., Simon S.M., Rappoport J.Z. (2010). Imaging with total internal reflection fluorescence microscopy for the cell biologist. J. Cell Sci..

[16] Vicidomini G., Bianchini P., Diaspro A. (2018). STED super-resolved microscopy. Nat. Methods.

[17] Blom H., Widengren J. (2017). Stimulated Emission Depletion Microscopy. Chem. Rev..

[18] Xu R., Xu Y., Wang Z., Zhou Y., Dang D., Meng L. (2021). Recent Advances on Organic Fluorescent Probes for Stimulated Emission Depletion (STED) Microscopy. Comb. Chem. High. Throughput Screen..

[19] Wu Z., Xu X., Xi P. (2021). Stimulated emission depletion microscopy for biological imaging in four dimensions: A review. Microsc. Res. Tech..

[20] Strack R. (2018). Hessian structured illumination microscopy. Nat. Methods.

[21] Temma K., Oketani R., Kubo T., Bando K., Maeda S., Sugiura K., Matsuda T., Heintzmann R., Kaminishi T., Fukuda K. (2024). Selective-plane-activation structured illumination microscopy. Nat. Methods.

[22] Hirano Y., Matsuda A., Hiraoka Y. (2015). Recent advancements in structured-illumination microscopy toward live-cell imaging. Microscopy.

[23] Mo Y., Feng F., Mao H., Fan J., Chen L. (2021). Structured illumination microscopy artefacts caused by illumination scattering. Philos. Trans. A Math. Phys. Eng. Sci..

[24] Xu J., Ma H., Liu Y. (2017). Stochastic Optical Reconstruction Microscopy (STORM). Curr. Protoc. Cytom..

[25] Huang B., Wang W.Q., Bates M., Zhuang X.W. (2008). Three-dimensional super-resolution imaging by stochastic optical reconstruction microscopy. Science.

[26] Rust M.J., Bates M., Zhuang X. (2006). Sub-diffraction-limit imaging by stochastic optical reconstruction microscopy (STORM). Nat. Methods.

[27] Tam J., Merino D. (2015). Stochastic optical reconstruction microscopy (STORM) in comparison with stimulated emission depletion (STED) and other imaging methods. J. Neurochem..

[28] Chambers M.G., McNamara R.P., Dittmer D.P. (2021). Direct Stochastic Optical Reconstruction Microscopy of Extracellular Vesicles in Three Dimensions. J. Vis. Exp..

[29] Jensen L.G., Hoh T.Y., Williamson D.J., Griffié J., Sage D., Rubin-Delanchy P., Owen D.M. (2022). Correction of multiple-blinking artifacts in photoactivated localization microscopy. Nat. Methods.

[30] Sengupta P., van Engelenburg S.B., Lippincott-Schwartz J. (2014). Superresolution imaging of biological systems using photoactivated localization microscopy. Chem. Rev..

[31] Bayle V., Fiche J.B., Burny C., Platre M.P., Nollmann M., Martinière A., Jaillais Y. (2021). Single-particle tracking photoactivated localization microscopy of membrane proteins in living plant tissues. Nat. Protoc..

[32] Sauer M., Heilemann M. (2017). Single-Molecule Localization Microscopy in Eukaryotes. Chem. Rev..

[33] Descloux A., Grussmayer K.S., Bostan E., Lukes T., Bouwens A., Sharipov A., Geissbuehler S., Mahul-Mellier A.L., Lashuel H.A., Leutenegger M. (2018). Combined multi-plane phase retrieval and super-resolution optical fluctuation imaging for 4D cell microscopy. Nat. Photonics.

[34] Zeng Z., Ma J., Chen X., Xu C. (2019). Lifetime super-resolution optical fluctuation imaging. J. Microsc..

[35] Glogger M., Spahn C., Enderlein J., Heilemann M. (2021). Multi-Color, Bleaching-Resistant Super-Resolution Optical Fluctuation Imaging with Oligonucleotide-Based Exchangeable Fluorophores. Angew. Chem. Int. Ed. Engl..

[36] Grußmayer K., Lukes T., Lasser T., Radenovic A. (2020). Self-Blinking Dyes Unlock High-Order and Multiplane Super-Resolution Optical Fluctuation Imaging. ACS Nano.

[37] Brockman J.M., Su H.Q., Blanchard A.T., Duan Y.X., Meyer T., Quach M.E., Glazier R., Bazrafshan A., Bender R.L., Kellner A.V. (2020). Live-cell super-resolved PAINT imaging of piconewton cellular traction forces. Nat. Methods.

[38] Delcanale P., Miret-Ontiveros B., Arista-Romero M., Pujals S., Albertazzi L. (2018). Nanoscale Mapping Functional Sites on Nanoparticles by Points Accumulation for Imaging in Nanoscale Topography (PAINT). ACS Nano.

[39] Dai Z., Xie X., Gao Z., Li Q. (2022). DNA-PAINT Super-Resolution Imaging for Characterization of Nucleic Acid Nanostructures. Chempluschem.

[40] Chung K.K.H., Zhang Z., Kidd P., Zhang Y., Williams N.D., Rollins B., Yang Y., Lin C., Baddeley D., Bewersdorf J. (2022). Fluorogenic DNA-PAINT for faster, low-background super-resolution imaging. Nat. Methods.

[41] Chen F., Tillberg P.W., Boyden E.S. (2015). Optical imaging. Expansion microscopy. Science.

[42] Tillberg P.W., Chen F., Piatkevich K.D., Zhao Y., Yu C.C., English B.P., Gao L., Martorell A., Suk H.J., Yoshida F. (2016). Protein-retention expansion microscopy of cells and tissues labeled using standard fluorescent proteins and antibodies. Nat. Biotechnol..

[43] Wassie A.T., Zhao Y., Boyden E.S. (2019). Expansion microscopy: Principles and uses in biological research. Nat. Methods.

[44] Chang J.B., Chen F., Yoon Y.G., Jung E.E., Babcock H., Kang J.S., Asano S., Suk H.J., Pak N., Tillberg P.W. (2017). Iterative expansion microscopy. Nat. Methods.

[45] Otomo K., Hibi T., Kozawa Y., Nemoto T. (2015). STED microscopy–super-resolution bio-imaging utilizing a stimulated emission depletion. Microscopy.

[46] Zhou J., Chizhik A.I., Chu S., Jin D. (2020). Single-particle spectroscopy for functional nanomaterials. Nature.

[47] Jin D., Xi P., Wang B., Zhang L., Enderlein J., van Oijen A.M. (2018). Nanoparticles for super-resolution microscopy and single-molecule tracking. Nat. Methods.

[48] Xu Y., Xu R., Wang Z., Zhou Y., Shen Q., Ji W., Dang D., Meng L., Tang B.Z. (2021). Recent advances in luminescent materials for super-resolution imaging via stimulated emission depletion nanoscopy. Chem. Soc. Rev..

[49] Liu Y., Peng Z., Peng X., Yan W., Yang Z., Qu J. (2021). Shedding New Lights Into STED Microscopy: Emerging Nanoprobes for Imaging. Front. Chem..

[50] Li W., Kaminski Schierle G.S., Lei B., Liu Y., Kaminski C.F. (2022). Fluorescent Nanoparticles for Super-Resolution Imaging. Chem. Rev..

[51] Pramanik S.K., Sreedharan S., Tiwari R., Dutta S., Kandoth N., Barman S., Aderinto S.O., Chattopadhyay S., Das A., Thomas J.A. (2022). Nanoparticles for super-resolution microscopy: Intracellular delivery and molecular targeting. Chem. Soc. Rev..

[52] Arroyo-Camejo S., Adam M., Besbes M., Hugonin P., Jacques V., Greffet J., Roch J., Hell S., Treussart F. (2013). Stimulated emission depletion microscopy resolves individual nitrogen vacancy centers in diamond nanocrystals. ACS Nano.

[53] Hanne J., Falk H.J., Görlitz F., Hoyer P., Engelhardt J., Sahl S.J., Hell S.W. (2015). STED nanoscopy with fluorescent quantum dots. Nat. Commun..

[54] Liu Y., Lu Y., Yang X., Zheng X., Wen S., Wang F., Vidal X., Zhao J., Liu D., Zhou Z. (2017). Amplified stimulated emission in upconversion nanoparticles for super-resolution nanoscopy. Nature.

[55] Li H., Zhang W., Bian Y., Ahn T.K., Shen H., Ji B. (2022). ZnF(2)-Assisted Synthesis of Highly Luminescent InP/ZnSe/ZnS Quantum Dots for Efficient and Stable Electroluminescence. Nano Lett..

[56] Zhou J., Yang Y., Zhang C.Y. (2015). Toward Biocompatible Semiconductor Quantum Dots: From Biosynthesis and Bioconjugation to Biomedical Application. Chem. Rev..

[57] Bilan R., Fleury F., Nabiev I., Sukhanova A. (2015). Quantum dot surface chemistry and functionalization for cell targeting and imaging. Bioconjug. Chem..

[58] Yang X., Zhang K., Wang H., Liu Y., Wang F., Zhang X., Shi K., Gao J., Jin D., Xi P. (2016). Versatile Application of Fluorescent Quantum Dot Labels in Superresolution Fluorescence Microscopy. ACS Photonics.

[59] Zhou L., Yu B., Huang L., Cao H., Lin D., Jing Y., Wali F., Qu J. (2022). Nonblinking Core–Multishell InP/ZnSe/ZnS Quantum Dot Bioconjugates for Super-resolution Imaging. ACS Appl. Nano Mater..

[60] Ye S., Yan W., Zhao M., Peng X., Song J., Qu J. (2018). Low-Saturation-Intensity, High-Photostability, and High-Resolution STED Nanoscopy Assisted by CsPbBr(3) Quantum Dots. Adv. Mater..

[61] Ye S., Guo J., Song J., Qu J. (2020). Achieving high-resolution of 21 nm for STED nanoscopy assisted by CdSe@ZnS quantum dots. Appl. Phys. Lett..

[62] Bai X., Wang K., Chen L., Zhou J., Wang J. (2022). Semiconducting polymer dots as fluorescent probes for in vitro biosensing. J. Mater. Chem. B..

[63] Wu Y., Shi C., Wang G., Sun H., Yin S. (2022). Recent advances in the development and applications of conjugated polymer dots. J. Mater. Chem. B..

[64] Yuan Y., Hou W., Qin W., Wu C. (2021). Recent advances in semiconducting polymer dots as optical probes for biosensing. Biomater. Sci..

[65] Wu C., Schneider T., Zeigler M., Yu J., Schiro P.G., Burnham D.R., McNeill J.D., Chiu D.T. (2010). Bioconjugation of ultrabright semiconducting polymer dots for specific cellular targeting. J. Am. Chem. Soc..

[66] Wu Y., Ruan H., Zhao R., Dong Z., Li W., Tang X., Yuan J., Fang X. (2018). Ultrastable Fluorescent Polymer Dots for Stimulated Emission Depletion Bioimaging. Adv. Opt. Mater..

[67] Wu Y., Ruan H., Dong Z., Zhao R., Yu J., Tang X., Kou X., Zhang X., Wu M., Luo F. (2020). Fluorescent Polymer Dot-Based Multicolor Stimulated Emission Depletion Nanoscopy with a Single Laser Beam Pair for Cellular Tracking. Anal. Chem..

[68] Xin N., Gao D., Su B., Zhou T., Zhu Y., Wu C., Wei D., Sun J., Fan H. (2023). Orange-Emissive Carbon Dots with High Photostability for Mitochondrial Dynamics Tracking in Living Cells. ACS Sens..

[69] Ma W., Wang B., Yang Y., Li J. (2021). Photoluminescent chiral carbon dots derived from glutamine. Chin. Chem. Lett..

[70] Liu Y., Yu N., Fang W., Tan Q., Ji R., Yang L., Wei S., Zhang X., Miao A. (2021). Photodegradation of carbon dots cause cytotoxicity. Nat Commun..

[71] He H., Liu X., Li S., Wang X., Wang Q., Li J., Wang J., Ren H., Ge B., Wang S. (2017). High-Density Super-Resolution Localization Imaging with Blinking Carbon Dots. Anal. Chem..

[72] Lemenager G., De Luca E., Sun Y.P., Pompa P.P. (2014). Super-resolution fluorescence imaging of biocompatible carbon dots. Nanoscale.

[73] Li H., Ye S., Guo J., Wang H., Yan W., Song J., Qu J. (2019). Biocompatible carbon dots with low-saturation-intensity and high-photobleaching-resistance for STED nanoscopy imaging of the nucleolus and tunneling nanotubes in living cells. Nano Res..

[74] Han G., Zhao J., Zhang R., Tian X., Liu Z., Wang A., Liu R., Liu B., Han M.Y., Gao X. (2019). Membrane-Penetrating Carbon Quantum Dots for Imaging Nucleic Acid Structures in Live Organisms. Angew. Chem. Int. Ed. Engl..

[75] Hua X.W., Bao Y.W., Zeng J., Wu F.G. (2019). Nucleolus-Targeted Red Emissive Carbon Dots with Polarity-Sensitive and Excitation-Independent Fluorescence Emission: High-Resolution Cell Imaging and in Vivo Tracking. ACS Appl. Mater. Interfaces.

[76] Yang J., Zhang X., Ma Y., Gao G., Chen X., Jia H., Li Y., Chen Z., Wu F. (2016). Carbon Dot-Based Platform for Simultaneous Bacterial Distinguishment and Antibacterial Applications. ACS Appl. Mater. Interfaces.

[77] De Camillis S., Ren P., Cao Y., Ploschner M., Denkova D., Zheng X., Lu Y., Piper J.A. (2020). Controlling the non-linear emission of upconversion nanoparticles to enhance super-resolution imaging performance. Nanoscale.

[78] Wu Q., Huang B., Peng X., He S., Zhan Q. (2017). Non-bleaching fluorescence emission difference microscopy using single 808-nm laser excited red upconversion emission. Opt. Express.

[79] Huang B., Wu Q., Peng X., Yao L., Peng D., Zhan Q. (2018). One-scan fluorescence emission difference nanoscopy developed with excitation orthogonalized upconversion nanoparticles. Nanoscale.

[80] Kim J., Kwon J.H., Jang J., Lee H., Kim S., Hahn Y.K., Kim S.K., Lee K.H., Lee S., Pyo H. (2018). Rapid and background-free detection of avian influenza virus in opaque sample using NIR-to-NIR upconversion nanoparticle-based lateral flow immunoassay platform. Biosens. Bioelectron..

[81] Li S., Song X., Zhu W., Chen Y., Zhu R., Wang L., Chen X., Song J., Yang H. (2020). Light-Switchable Yolk-Mesoporous Shell UCNPs@MgSiO(3) for Nitric Oxide-Evoked Multidrug Resistance Reversal in Cancer Therapy. ACS Appl. Mater. Interfaces.

[82] Gao P., Prunsche B., Zhou L., Nienhaus K., Nienhaus G.U. (2017). Background suppression in fluorescence nanoscopy with stimulated emission double depletion. Nat. Photonics.

[83] Chen C., Wang F., Wen S., Su Q.P., Wu M.C.L., Liu Y., Wang B., Li D., Shan X., Kianinia M. (2018). Multi-photon near-infrared emission saturation nanoscopy using upconversion nanoparticles. Nat. Commun..

[84] Zhan Q., Liu H., Wang B., Wu Q., Pu R., Zhou C., Huang B., Peng X., Agren H., He S. (2017). Achieving high-efficiency emission depletion nanoscopy by employing cross relaxation in upconversion nanoparticles. Nat. Commun..

[85] Chen C.H., Liu B.L., Liu Y.T., Liao J.Y., Shan X.C., Wang F., Jin D.Y. (2021). Heterochromatic Nonlinear Optical Responses in Upconversion Nanoparticles for Super-Resolution Nanoscopy. Adv. Mater..

[86] Liu Y., Wen S., Wang F., Zuo C., Chen C., Zhou J., Jin D. (2023). Population Control of Upconversion. Energy Transfer for Stimulation Emission Depletion Nanoscopy. Adv. Sci..

[87] Chen C., Ni X., Tian H.W., Liu Q., Guo D.S., Ding D. (2020). Calixarene-Based Supramolecular AIE Dots with Highly Inhibited Nonradiative Decay and Intersystem Crossing for Ultrasensitive Fluorescence Image-Guided Cancer Surgery. Angew. Chem. Int. Ed. Engl..

[88] Dang D., Zhang H., Xu Y., Xu R., Wang Z., Kwok R.T.K., Lam J.W.Y., Zhang L., Meng L., Tang B.Z. (2019). Super-Resolution Visualization of Self-Assembling Helical Fibers Using Aggregation-Induced Emission Luminogens in Stimulated Emission Depletion Nanoscopy. ACS Nano.

[89] Fang X., Chen X., Li R., Liu Z., Chen H., Sun Z., Ju B., Liu Y., Zhang S.X., Ding D. (2017). Multicolor Photo-Crosslinkable AIEgens toward Compact Nanodots for Subcellular Imaging and STED Nanoscopy. Small.

[90] Xu R., Dang D., Wang Z., Zhou Y., Xu Y., Zhao Y., Wang X., Yang Z., Meng L. (2022). Facilely prepared aggregation-induced emission (AIE) nanocrystals with deep-red emission for super-resolution imaging. Chem. Sci..

[91] Cao S., Tian X., Cao M., Wang J., Niu G., Tang B.Z. (2023). Solvatochromic Near-Infrared Aggregation-Induced Emission-Active Acrylonitriles by Acceptor Modulation for Low-Power Stimulated Emission Depletion Nanoscopy. Chem. Mater..

[92] Li D., Qin W., Xu B., Qian J., Tang B.Z. (2017). AIE Nanoparticles with High Stimulated Emission Depletion Efficiency and Photobleaching Resistance for Long-Term Super-Resolution Bioimaging. Adv. Mater..

[93] Pyo K., Thanthirige V.D., Kwak K., Pandurangan P., Ramakrishna G., Lee D. (2015). Ultrabright Luminescence from Gold Nanoclusters: Rigidifying the Au(I)–Thiolate Shell. J. Am. Chem. Soc..

[94] Goswami N., Zheng K., Xie J. (2014). Bio-NCs—The marriage of ultrasmall metal nanoclusters with biomolecules. Nanoscale.

[95] Chen Y., Phipps M.L., Werner J.H., Chakraborty S., Martinez J.S. (2018). DNA Templated Metal Nanoclusters: From Emergent Properties to Unique Applications. Acc. Chem. Res..

[96] Yang H., Wu Y., Ruan H., Guo F., Liang Y., Qin G., Liu X., Zhang Z., Yuan J., Fang X. (2022). Surface-Engineered Gold Nanoclusters for Stimulated Emission Depletion and Correlated Light and Electron Microscopy Imaging. Anal. Chem..

[97] Qi L., Xiao Y., Fu X., Yang H., Fang L., Xu R., Ping J., Han D., Jiang Y., Fang X. (2024). Monodispersed and Monofunctionalized DNA-Caged Au Nano-Clusters with Enhanced Optical Properties for STED Imaging. Small.

[98] Wang L., Ta H., Ullal C., Wang F., Wang C., Dong G. (2017). Aptamer functionalized silver clusters for STED microscopy. RSC Adv..

[99] Zhang B., Fang C.Y., Chang C.C., Peterson R., Maswadi S., Glickman R.D., Chang H.C., Ye J.Y. (2012). Photoacoustic emission from fluorescent nanodiamonds enhanced with gold nanoparticles. Biomed. Opt. Express.

[100] Liu Y., Ding Y., Alonas E., Zhao W., Santangelo P.J., Jin D., Piper J.A., Teng J., Ren Q., Xi P. (2012). Achieving lambda/10 resolution CW STED nanoscopy with a Ti:Sapphire oscillator. PLoS ONE.

[101] Tzeng Y.K., Faklaris O., Chang B.M., Kuo Y., Hsu J.H., Chang H.C. (2011). Superresolution imaging of albumin-conjugated fluorescent nanodiamonds in cells by stimulated emission depletion. Angew. Chem. Int. Ed. Engl..

[102] Han K.Y., Willig K.I., Rittweger E., Jelezko F., Eggeling C., Hell S.W. (2009). Three-dimensional stimulated emission depletion microscopy of nitrogen-vacancy centers in diamond using continuous-wave light. Nano Lett..

[103] Laporte G., Psaltis D. (2016). STED imaging of green fluorescent nanodiamonds containing nitrogen-vacancy-nitrogen centers. Biomed. Opt. Express.

[104] Prabhakar N., Peurla M., Koho S., Deguchi T., Nareoja T., Chang H.C., Rosenholm J.M., Hanninen P.E. (2018). STED-TEM Correlative Microscopy Leveraging Nanodiamonds as Intracellular Dual-Contrast Markers. Small.

[105] Man Z., Cui H., Lv Z., Xu Z., Wu Z., Wu Y., Liao Q., Liu M., Xi P., Zheng L. (2021). Organic Nanoparticles-Assisted Low-Power STED Nanoscopy. Nano Lett..

[106] Shang L., Gao P., Wang H., Popescu R., Gerthsen D., Nienhaus G.U. (2017). Protein-based fluorescent nanoparticles for super-resolution STED imaging of live cells. Chem. Sci..

[107] Man Z.W., Lv Z., Xu Z.Z., Yao J.N., Fu H.B. (2021). Strategic Engineering of Sub-5 nm Dyes@CDs Nanoassemblies Platform for Super Resolution Imaging. Adv. Funct. Mater..

[108] Peuschel H., Ruckelshausen T., Cavelius C., Kraegeloh A. (2015). Quantification of Internalized Silica Nanoparticles via STED Microscopy. Biomed Res. Int..

[109] Xu Y., Zhang H., Zhang N., Wang X., Dang D., Jing X., Xi D., Hao Y., Tang B., Meng L. (2020). Deep-Red Fluorescent Organic Nanoparticles with High Brightness and Photostability for Super-Resolution in Vitro and in Vivo Imaging Using STED Nanoscopy. ACS Appl. Mater. Interfaces.

